# Combined effect of pegylated interferon α with adefovir on renal function in Chinese patients with chronic hepatitis B

**DOI:** 10.1097/MD.0000000000012089

**Published:** 2018-08-24

**Authors:** Qian Su, Yanyan Liu, Jiabin Li

**Affiliations:** a Department of Infectious Diseases, First Affiliated Hospital of Anhui Medical University; b Department of Infectious Diseases, Chaohu Affiliated Hospital of Anhui Medical University, Hefei, China.

**Keywords:** adefovir, combination therapy, glomerular filtration rate, interferon, renal function

## Abstract

**Background:**

Long-term safety of treatment with hepatitis B virus (HBV) polymerase inhibitors is a concern. Adefovir dipivoxil (ADV) and/or interferon alfa (IFN-α) therapies have previously been associated with impairment of renal function. Limited data are available on the safety of combination therapy with nucleos (t)ide analogues (NAs) and IFN-α. The aim of this analysis was to assess the renal function during combined therapy with pegylated interferon α-2b (PEG-IFN-α-2b) and ADV versus PEG-IFN-α-2b alone in patients with chronic hepatitis B (CHB).

**Methods:**

We performed a multicenter, prospective, open-label, randomized-controlled trial of renal function data to investigate the efficacy of 48 weeks of therapy with PEG-IFN-α-2b and ADV versus PEG-IFN-α-2b alone in 102 patients with CHB in Anhui, China. Glomerular filtration rates (GFRs) were calculated by Cockcroft–Gault (CG), abbreviated Modification of Diet in Renal Disease (MDRD) study, and Chronic Kidney Disease Epidemiology Collaboration (CKD-EPI) equation, and were tested by repeated-measures 1-way analysis of variance within groups. A linear mixed effects model for repeated measures was also used to evaluate the association between baseline information and estimated glomerular filtration rate (eGFR) changes overtime in all enrolled patients. The model considered the baseline age, sex, HBV DNA, aminotransferase, treatment group, time, and group-by-time interaction as fixed effects and incorporated random effects for individual subjects.

**Results:**

After 48 weeks of therapy and further 24 weeks of follow-up, the eGFR decreased both in patients given PEG-IFN-α-2b single therapy and combined therapy. Age, HBV DNA, and combined therapy were significant negative predictive factors for eGFR changes.

**Conclusion:**

The incidence of renal adverse events in both groups was low, and the combination therapy may have delayed, but reversible renal impairment.

## 1 Introduction

Hundreds of million people are carriers with hepatitis B surface antigen (HBsAg) and almost 80% of chronic hepatitis B (CHB) reside in the Asia region. Although highly effectively and safe vaccines have been used more than 30 years, hepatitis B virus (HBV) infection remains one of the most common causes of death resulting from liver diseases around the world. Besides, with higher numbers of CHB now being treated, several possible adverse effects have drawn more attention. One area of concern is renal function and a close association has been reported between CHB and chronic renal disease.^[1]^ Chronic HBV infection can cause renal dysfunction through immune complex mediated glomerular diseases, such as membranous nephropathy.^[2]^ In countries with endemic HBV infection, HBV-related glomerulopathies are an important cause of end-stage renal disease and renal replacement therapy.^[3]^

Renal function, monitored by the estimated glomerular filtration rate (eGFR) and the determination of creatinine (Cr), is frequently impaired in patients with compensated CHB. The European Virgil database in 24 European centers suggested that 15% and 4% of 381 CHB patients had eGFR at 50 to 80 or <50 mL/min before start of antiviral therapy, respectively.^[4]^ In a German cohort including CHB patients, 20 of 60 patients had chronic kidney disease (CKD) stage 2 (eGFR at 60–89 mL/min) before starting antiviral treatment.^[5]^ In addition, 35% to 45% had chronic kidney disease stage 2 at baseline among a cohort study performed in 290 CHB Asian patients living in the United States.^[6]^ Renal dysfunction can also develop in patients with CHB with advanced/end-stage liver disease or decompensated cirrhosis through multiple mechanisms, including functional renal insufficiency (hepatorenal syndrome). In patients with decompensated CHB, creatinine clearance (Ccr) <70 mL/min was observed in 33% of patients with end-stage liver disease and renal function impairment was shown to be correlated with impaired liver function and mortality rates.^[2,7,8]^

Well-accepted guidelines for the management of HBV infection have been established in recent years. Currently approved treatments for CHB include 2 categories of drugs: (1) immune modulators such as pegylated interferon-alpha (PEG-IFN-α), and (2) nucleoside analogs (lamivudine, entecavir, and telbivudine) and nucleotide analogs (adefovir dipivoxil and tenofovir disoproxil fumarate), which suppress viral replication by selectively inhibiting the activity of HBV polymerase.^[9]^ Although nucleos (t)ide analogues (NAs) are cheap and safe, their limited long-term efficacy results in prolonged treatment periods, particularly in patients with HBeAg-negative CHB. Extended use of NAs has been shown to be associated with the generation of drug-resistant mutations.^[10]^ However, the duration of interferon-based therapy is predetermined and finite. The ultimate objective of HBV treatment should be loss of HBsAg with development of anti-HBs (HBsAg seroconversion), which, however, is only achieved in <10% of patients treated with IFN-α and in a small subgroup of patients treated with the current routine therapies.^[11]^ Some studies have combined the immunomodulatory properties of PEG-IFN along with the direct antiviral activity of NAs in an attempt to improve therapeutic efficacy in CHB patients.^[12–14]^ Patients treated with a combination of PEG-IFN-α and ADV had a higher on-treatment virological response (VR) rate than patients treated with IFN-α monotherapy.^[15]^ On the basis of the previous studies, Huang et al^[16]^ performed a meta-analysis and found that the efficacy of IFN-α and ADV combination therapy is superior to IFN-α monotherapy. However, few studies focus on the safe renal profile of combination of IFN-α and ADV, especially PEG-IFN-α-2b (PEG-IFN-α-2b), which was recommended as first-line antiviral drugs by National Institute for Health and Clinical Excellence. Hence, the aim of this randomized controlled trial was to assess the renal function and antiviral efficacy under IFN-α and ADV combined therapy and/or IFN-α monotherapy in CHB patients. Known risk factors were also took into account to analyze the predictors for significant eGFR change.

## 2 Methods

### 2.1 Participants and study design

We did a prospective, multicenter, open-label, randomized trial in 10 hospitals (First Affiliated Hospital of Anhui Medical University, Chaohu Affiliated Hospital of Anhui Medical University, First Affiliated Hospital of Anhui University of Chinese Medicine, Second People's Hospital of Fuyang City, First Affiliated Hospital of Wannan Medical College, People's Hospital of Chizhou City, People's Hospital of Xuancheng City, Huainan Eastern Hospital, the 105 Hospital of People's Liberation Army, and Central Hospital of Xuancheng City) in Anhui, China, between June 2012 and September 2013. The study was conducted in accordance with the guidelines of the Declaration of Helsinki and the principles of Good Clinical Practice and China regulatory requirements and was approved by the local Ethics Committees of the First Affiliated Hospital of Anhui Medical University, Hefei, China (No. K2010003). All patients had written informed consent. The registration number of Clinical Trials was ChiCTR-TRC-12002226. We recruited consecutive patients with newly diagnosed CHB aged between 18 and 65 years if they fulfilled the following criteria: diagnoses of CHB according to the standard of the Chinese National Program for Prevention and Treatment of Viral Hepatitis; HBeAg-positive and HBsAg-positive patients with elevated ALT levels of at least 2 upper limit of normal with serum HBV DNA levels more than 2 × 10^4^ IU/mL; absence of other hepatitis virus or HIV coinfection; and absence of concurrently afflicted by decompensated liver cirrhosis (including ascites, hepatic encephalopathy, variceal bleeding, spontaneous bacterial peritonitis), liver failure, or hepatocellular carcinoma; absence of hypertension, diabetes mellitus, immunocompromised diseases, autoimmune diseases, solid cancer, or leukemia. All patients were treated for the first time, and none was recruited into the study at the time of a relapse. We randomly assigned participants (1:1) to either a PEG-IFN-α-2b treatment alone (1.5 μg/kg, subcutaneous injection weekly), or PEG-IFN-α-2b (1.5 μg/kg, subcutaneous injection weekly) and ADV (10 mg, oral administration daily). As the medicines differed between the 2 groups, the study was not masked. We assigned treatment through central computer-generated randomization, with stratification on disease severity (severe or moderate). The sequence was generated by computer randomly. All patients included in this cohort underwent a follow-up evaluation at baseline, at week 0, 4, 8, 12, 24, 36, 48, and 72 for a total of 72 weeks. Virological and biochemical assessments were performed as routine examination at every visit.

### 2.2 Virological and biochemical assessment

Serum HBV DNA was quantified by real-time polymerase chain reaction kit Roche COBAS TaqMan (Roche Molecular Systems, Inc., Branchburg, NJ) with detection limit threshold of 20 IU/mL. HBsAg and HBeAg were quantified using the HBsAg and HBeAg reagent kit (Roche Molecular Systems, Inc., Branchburg, NJ), respectively. Serum biochemical assessments [including alanine aminotransferase (ALT), aspartate aminotransferase (AST)] and serum Cr were measured by biochemistry auto analyzer (Roche Cobas 8000; Roche Diagnostics GmbH, Mannheim, Germany) in the Department of Laboratory Medicine, the First Affiliated Hospital of Anhui Medical University. VR is defined as an HBV DNA concentration of less than 300 IU/mL after 48 weeks of treatment and biochemical response (BR) is defined as normalization of ALT levels after 48 weeks of treatment.

### 2.3 Evaluation of renal function

The Cockcroft–Gault (CG) equation relies on serum Cr, age, and body weight and is given as: Ccr = [140 -age (years)] × [body weight (kg)]/72 × Serum Cr (mg/dL) (15% less in women).^[17]^ The CG formula has been widely used in pharmacokinetic studies and particularly in defining drug dosing in patients with impaired renal function. The eGFR was estimated by the following formulas based on Cr. The Chronic Kidney Disease Epidemiology Collaboration (CKD-EPI) calculation for eGFR (mL/min/1.73 m^2^) = 141 × min (Cr/κ, L)^α^ × max (Cr/κ, 1)^−1.209^ × 0.993^Age^ × 1.018 (if female).^[18]^ κ is 0.7 for female and 0.9 for male. α is − 0.329 for female and −0.411 for male. The Modification of Diet in Renal Disease (MDRD) calculation for eGFR (mL/min/1.73 m^2^) = 175 × Cr^−1.154^ × Age^−0.203^ × 0.742 (if female).^[19]^

### 2.4 Statistical analysis

We used the SPSS16.0 software for generating randomized subject sequence. Patients’ demographic and baseline characteristic data were presented as mean ± standard deviations (SD) and median (IQR: 1st and 3rd quartiles) for continuous data with and without normal distribution; categorical data were presented as n (%). The Chi-squared test, independent *t* test, or Kruskal–Wallis test was used to assess the differences in demographic and clinical variables among groups. All continuous variables were tested by repeated-measures analysis of variance (ANOVA), which was used by R Version 3.3.3 with MIXED procedure. To evaluate the association between several variables and eGFR changes over time, a linear mixed effects model for repeated measures was used by R Version 3.3.3 with MIXED procedure. The model considered the baseline age (in years), sex, HBV DNA, ALT, AST, treatment group, time and group-by-time interaction as fixed effects, and incorporated random effects for individual subjects. All *P* values are 2-sided, and the type I error was set as 5%.

## 3 Results

### 3.1 Baseline characteristics of enrolled patients

The study was conducted between June 2012 and September 2013. A total of 2837 patients with a confirmed diagnosis of chronic hepatitis B carrier were screened. Among them, 102 patients met the inclusion criteria and were randomized to receive either PEG-IFN-α-2b treatment (51 patients) or PEG-IFN-α-2b and ADV (51 patients) treatment. The baseline demographic and clinical characteristics of the 2 groups are similar and summarized in Table 1. There was no significant difference in the age, gender ratio, ALT levels, and virological baseline characteristics between the 2 cohorts.

**Table 1:**
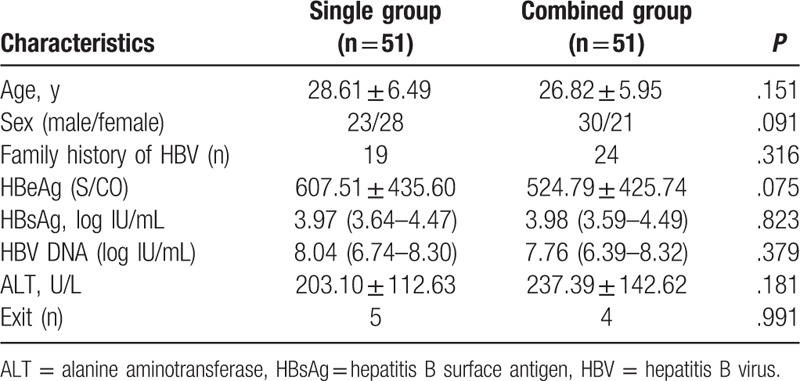
Baseline characteristics of the patients.

**Figure 1:**
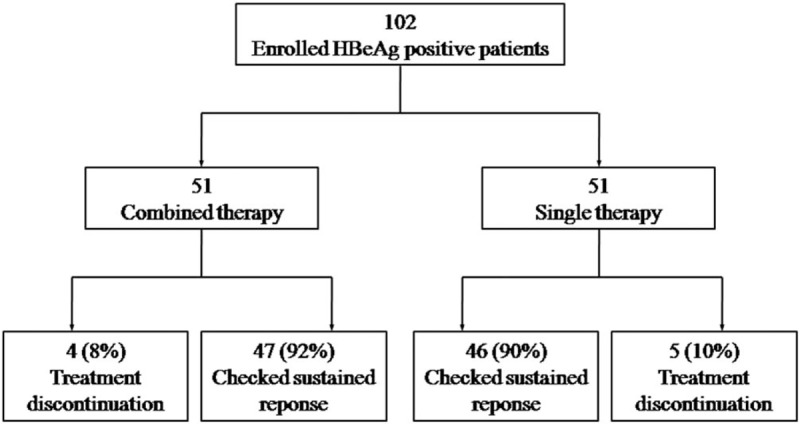
Patients’ flow sheet.

Review of the patient charts indicated that most of recruited patients had good compliance. However, during treatment, 9 patients were lost, including 4 who were treated with combined treatment and 5 in the single group. The common reason for discontinuing treatment was withdrawal of consent and lost to follow-up (details shown in Fig. 1). Thus, 47 (92%) patients in the combined treatment group and 46 (90%) patients in the single group completed the 72 weeks of follow-up (Fig. 1).

### 3.2 Virological, biochemical, and serological responses

**Figure 2:**
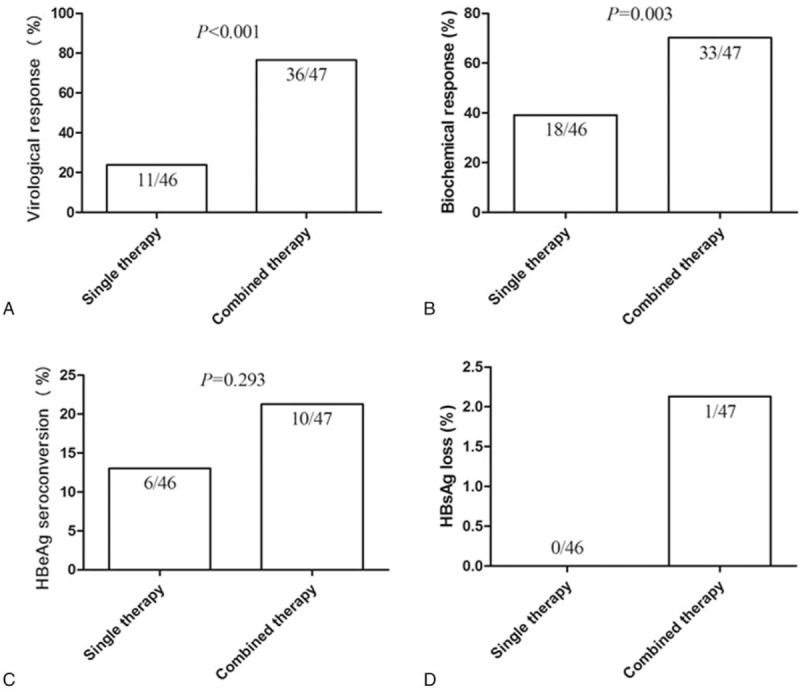
The rates corresponding to virological, biochemical, and serological responses to anti-HBV agents. (A) Rate of virological response at 72 weeks of therapy. (B) Rate of biochemical response (ALT normalization) at 72 weeks of therapy. (C) Rate of serologic response (HBeAg/anti-HBe seroconversion) at 72 weeks of therapy. (D) Rate of serologic response (HBsAg loss) at 72 weeks of therapy. HBsAg = hepatitis B surface antigen.

The HBV DNA decreased in CHB patients received anti-HBV therapy. Greater proportions of patients with PEG-IFN-α-2b and ADV combined therapy (76.6%) showed significantly higher VRs than single PEG-IFN-α-2b (23.9%) at 48 weeks of therapy (*P* < .001, Fig. 2A). Eighteen single-experienced patients and 33 combined-experienced patients who revealed elevated ALT at baseline achieved BR with normal ALT levels at 48 weeks of therapy, respectively. Furthermore, combined therapy (70.2%) revealed remarkable higher BR rates than patients who received PEG-IFN-α-2b (39.1%) at 48 weeks of therapy (*P* = .003, Fig. 2B). Moreover, greater proportions of patients who received combined therapy showed higher HBeAg seroconversion rate than single therapy at week 48 (*P* = .293, Fig. 2C). Namely, 6 and 10 patients with single therapy and combined therapy revealed HBeAg/anti-HBe seroconversion, respectively. However, HBsAg loss was not observed in PEG-IFN-α-2b treated patients. One patient with combined therapy demonstrated HBsAg loss during therapy (Fig. 2D).

### 3.3 Maintenance of renal function in different therapies for CHB

**Figure 3:**
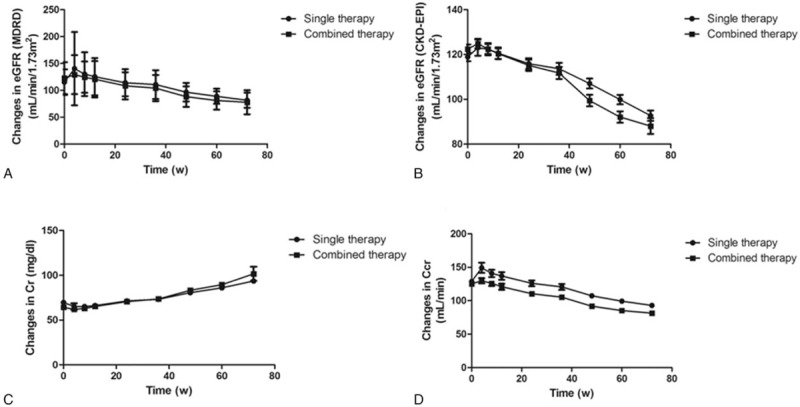
Evolution of renal function by anti-HBV agents therapy over 72 weeks. (A) Changes of eGFR as calculated by MDRD formula. (B) Changes of eGFR as calculated by CKD-EPI formula. (C) Changes of serum Cr. (D) Changes of serum Ccr.

On the basis of the MDRD formula, a total of 68 patients (36 receiving combined therapy and 32 receiving single therapy) demonstrated renal dysfunction after antiviral therapy. The changes in renal function (including Cr, Ccr, and eGFR) were evaluated using repeated-measures ANOVA, which represented the matched values in different time points (Fig. 3). Results with CKD-EPI and MDRD equations were comparable for eGFR changes during the 48 weeks of therapy and further 24 weeks of follow-up. There were also no significant differences in Ccr and Cr during either combined or single treatment during the 72 weeks of follow-up. Interestingly, renal function improved first at week 4 [eGFR (CKD-EPI) changes: +15.7 mL/min/1.73 m^2^; eGFR (MDRD) changes: +3.3 mL/min/1.73 m^2^; Ccr changes: +12.4 mL/min], and then steadily declined in all 93 patients with both PEG-IFN-α-2b therapy and combined therapy. eGFR of 2 groups achieved 79.7 ± 18.7 mL/min/1.73 m^2^ (CKD-EPI) and 90.0 ± 19.8 mL/min/1.73 m^2^ (MDRD) at the end of antiviral therapy, respectively. Both serum Cr and Ccr revealed similar decreased trends with eGFR changes.

### 3.4 Predictors of significant eGFR change

We entered all variables, including HBV DNA, ADV-containing treatment, and group-by-time interaction as fixed effects and incorporated random effects in the linear mixed model accounting for repeated measures. PEG-IFN-α-2b therapy was set as reference in this model. Results with CKD-EPI and MDRD equations were also comparable for the predictors of eGFR changes. We found that age, HBV DNA, and ADV administration were significant predictors for decreased eGFR over time. Among these variables, ADV administration was most capable of predicting eGFR decreases in CHB patients [estimated value of -3.218 (CKD-EPI) or -3.852 (MDRD), *P* < .001]. Interestingly, sex was observed to be positively influenced eGFR values overtime without statistically significant [estimated value of 0.609 (CKD-EPI) or 0.677 (MDRD), *P* = .617 and = .523, respectively]. Furthermore, the changes of eGFR over time were not significantly associated with the reduction in both time and aminotransferase (details shown inTable 2).

**Table 2:**
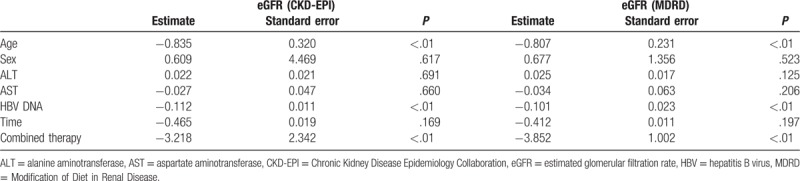
Predictors of eGFR changes.

## 4 Discussion

The current study was designed to evaluate renal function of CHB patients who were treated with PEG-IFN-α-2b and ADV or PEG-IFN-α-2b alone. We here suggested that both combined therapy and single PEG-IFN-α-2b treatment were associated with significant decreases in eGFR of patients with hepatitis B infection. This impairment was in a similar range to previous published data on HBV/D virus coinfection.^[20]^ Lamivudine, entecavir, telbivudine, and ADV, the HBV polymerase inhibitors, have been approved for the CHB for over a decade. However, all of these antiviral agents have a low activity against the human mitochondrial DNA polymerase gamma, which could damage mitochondrial replication, resulting in mitochondrial dysfunction or loss and potentially leading to clinical adverse events, such as myopathy, neuropathy, and/or lactic acidosis.^[21]^ Previous studies suggested that combined therapy with telbivudine and PEG-IFN-α was related with unexpected cases of neuropathy.^[22]^ However, PEG-IFN-α and lamivudine seemed to be safe and effective.^[14]^ In the present study, our results are of importance that the combination of ADV and PEG-IFN-α-2b did not cause any further renal impairment. The safety of PEG-IFN-α-2b/ADV combination might be of especial interest, as the combined therapy could lead to a more pronounced effect in decreased HBsAg in CHB patients.

To the best of our knowledge, this is the first study on the impairment of renal function with PEG-IFN-α-2b plus ADV therapy for HBV monoinfection. Our results were in line with a previous study showing a decrease in eGFR for patients with hepatitis B/D virus coinfection who received 48-week PEG-IFN-α therapy.^[20]^ In addition, it has been generally elucidated that deposition of host antibodies and immune complexes of HBV antigens mediate most glomerular injuries.^[23]^ More recent studies on HBV-associated membranous nephropathy revealed that the CD^4+^CXCR^5+^ follicular T helper (Tfh) cells were negatively correlated with the range of eGFR.^[24]^ Li et al^[25]^ also indicated that circulating CD^4+^CXCR^5+^Tfh cells contributed to ADV-induced HBeAg seroconversion, which might indicate that the decrease in eGFR was a direct injury from PEG-IFN-α-2b itself rather than an indirect effect by suppression of viral replication. It has been confirmed that both PEG-IFN-α-2b and ADV showed immunomodulatory roles to regulate viral replication by activation of cellular and humoral immunity and suppression of negative regulators.^[26,27]^ Thus, we hypothesized that the immnunomodulatory properties of PEG-IFN-α-2b and ADV may partially lead to the decrease of eGFR. However, the specific mechanisms by which PEG-IFN-α-2b and ADV perform their renotoxic effects were still unclear and remain to be clarified in future studies.

When investigating potential predictors associated with the decrease of eGFR, we identified age, HBV DNA, and ADV-containing treatment as significant negative factors, consist with the previous studies in different ethnic origins. In the study of Mederacke et al,^[20]^ the majority of subjects were born either in the former Soviet Union or in Turkey, whereas most patients of present study were from Asia.^[22]^ The findings that older but not younger age and higher but not lower HBV DNA were associated with a higher risk to undergo a decline in eGFR during treatment were easy to understand.

Further studies on the changes in eGFR in patients with combination therapy of PEG-IFN-α-2b and NAs should be performed to investigate the predominant renoprotective or impairment effects of anti-HBV agents. This study has several limitations. Although results were derived from a prospective randomized controlled trial (RCT); this was only a retrospective analysis of renal injuries by eGFR values. The significant alteration of eGFR levels happened to be in the normal range and did not result in any clinical consequences in current study. The included patients were relatively young with most patients less than 60 years old. Furthermore, the limited number of included patients and relatively short observational time for 72 weeks may also represent restrictions of our study. Thus, a linear mixed effects model was conducted for repeated measure to evaluate the variations of eGFR in individuals. In future, we will continue to expand the sample content and continue to follow-up. Furthermore, no routine urine samples were collected during the study. Nucleotides (such as ADV) tend to be more harmful to tubular than glomerular cells in HBV infection. HBV infection could also induce subtle urinary abnormalities (e.g., proteinuria and hematuria) without obvious eGFR decrease in early stage. Levels of serum Cr and changes of eGFR are late markers of renal impairment, which presumably appear secondarily after proximal tubular dysfunction. Thus, the exact impact of specific tubular toxicity of anti-HBV agents cannot be reliably appreciated.

In conclusion, this study analyzing data from patients with HBV-infection confirms previous findings that the combination of ADV and PEG-IFN-α-2b and PEG-IFN-α-2b single therapy may indeed result in declines in GFR. But no difference between these 2 therapies on renal function was observed.

In the treatment of 48 and 12 weeks of withdrawal, the combined group may aggravate renal impairment, and the treatment with the ADV may have delayed but reversible renal toxicity. Therefore, regular monitoring of renal function and early identification can help intervene to prevent disease progression to end-stage renal disease.

## Author contributions

J.L. designed the research; Q.S. and Y.L. conducted the studies; Q.S. and Y.L. analyzed the data and prepared the manuscript; all authors read and approved the manuscript.

**Project administration:** Jiabin Li.

**Writing – original draft:** Qian Su, Yanyan Liu.

## References

[1] Ning L Lin W Hu X Prevalence of chronic kidney disease in patients with chronic hepatitis B: a cross-sectional survey. J Viral Hepat 2017;24:1043–51. 28581186 10.1111/jvh.12733

[2] Li Z Shen C Wang Y Circulating kidney injury molecule-1 is a novel diagnostic biomarker for renal dysfunction during long-term adefovir therapy in chronic hepatitis B. Medicine (Baltimore) 2016;95:e5264. 27858892 10.1097/MD.0000000000005264 PMC5591140

[3] Chan HL Shaikh J Gupta S Renal function in nucleos (t)ide analog-treated patients with chronic hepatitis B: a systematic literature review and network meta-analysis. Adv Ther 2016;33:862–75. 27146675 10.1007/s12325-016-0337-2 PMC4882346

[4] Gane EJ Deray G Liaw YF Telbivudine improves renal function in patients with chronic hepatitis B. Gastroenterology 2014;146:138–46. e5. 24067879 10.1053/j.gastro.2013.09.031

[5] Mauss S Berger F Filmann N Effect of HBV polymerase inhibitors on renal function in patients with chronic hepatitis B. J Hepatol 2011;55:1235–40. 21703180 10.1016/j.jhep.2011.03.030

[6] Ha NB Ha NB Garcia RT Medication nonadherence with long-term management of patients with hepatitis B e antigen-negative chronic hepatitis B. Dig Dis Sci 2011;56:2423–31. 21327918 10.1007/s10620-011-1610-5

[7] Fasano M Maggi P Leone A Long-term efficacy and safety of switching from lamivudine+adefovir to tenofovir disoproxil fumarate in virologically suppressed patients. Dig Liver Dis 2017;49:530–4. 28179096 10.1016/j.dld.2017.01.140

[8] Wang HM Hung CH Lee CM Three-year efficacy and safety of tenofovir in nucleos (t)ide analog-naïve and nucleos (t)ide analog-experienced chronic hepatitis B patients. J Gastroenterol Hepatol 2016;31:1307–14. 26758501 10.1111/jgh.13294

[9] European Association for the Study of the Liver. EASL 2017 Clinical Practice Guidelines on the management of hepatitis B virus infection. J Hepatol 2017;67:370–98. 28427875 10.1016/j.jhep.2017.03.021

[10] Liaw YF Chien RN Yeh CT B virus clearance after emergence of YMDD motif mutation during lamivudine therapy. Hepatology 1999;30:567–72. 10421670 10.1002/hep.510300221

[11] Hadziyannis AS Papaioannou C Spanou F Induction interferon therapy in naïve patients with chronic hepatitis C: increased end-of-treatment virological responses but absence of long-term benefit. Aliment Pharmacol Ther 2001;15:551–7. 11284785 10.1046/j.1365-2036.2001.00946.x

[12] Xu Y Wang X Liu Z Addition of nucleoside analogues to peg-IFN (-2a enhances virological response in chronic hepatitis B patients without early response to peg-IFN (-2a: a randomized controlled trial. BMC Gastroenterol 2017;17:102. 28854883 10.1186/s12876-017-0657-y PMC5577782

[13] Lau GK Piratvisuth T Luo KX Peginterferon Alfa-2a, lamivudine, and the combination for HBeAg-positive chronic hepatitis B. N Engl J Med 2005;352:2682–95. 15987917 10.1056/NEJMoa043470

[14] Marcellin P Lau GK Bonino F Peginterferon alfa-2a alone, lamivudine alone, and the two in combination in patients with HBeAg-negative chronic hepatitis B. N Engl J Med 2004;351:1206–17. 15371578 10.1056/NEJMoa040431

[15] Marcellin P Ahn SH Ma X Combination of tenofovir disoproxil fumarate and peginterferon (-2a increases loss of hepatitis B surface antigen in patients with chronic hepatitis B. Gastroenterology 2016;150:134–44. e10. 26453773 10.1053/j.gastro.2015.09.043

[16] Huang R Hao Y Zhang J Interferon-alpha plus adefovir combination therapy versus interferon-alpha monotherapy for chronic hepatitis B treatment: a meta-analysis. Hepatol Res 2013;43:1040–51. 23356962 10.1111/hepr.12058

[17] Cockcroft DW Gault MH Prediction of creatinine clearance from serum creatinine. Nephron 1976;16:31–41. 1244564 10.1159/000180580

[18] Levey AS Stevens LA Schmid CH A new equation to estimate glomerular filtration rate. Ann Intern Med 2009;150:604–12. 19414839 10.7326/0003-4819-150-9-200905050-00006 PMC2763564

[19] Levey AS Coresh J Greene T Expressing the Modification of Diet in Renal Disease Study equation for estimating glomerular filtration rate with standardized serum creatinine values. Clin Chem 2007;53:766–72. 17332152 10.1373/clinchem.2006.077180

[20] Mederacke I Yurdaydin C Großhennig A Renal function during treatment with adefovir plus peginterferon alfa-2a vs either drug alone in hepatitis B/D co-infection. J Viral Hepat 2012;19:387–95. 22571900 10.1111/j.1365-2893.2011.01560.x

[21] Lai KN Ho RT Tam JS Detection of hepatitis B virus DNA and RNA in kidneys of HBV related glomerulonephritis. Kidney Int 1996;50:1965–77. 8943480 10.1038/ki.1996.519

[22] Chen YC Su YC Li CY A nationwide cohort study suggests chronic hepatitis B virus infection increases the risk of end-stage renal disease among patients in Taiwan. Kidney Int 2015;87:1030–8. 25426815 10.1038/ki.2014.363

[23] Bhimma R Coovadia HM Hepatitis B virus-associated nephropathy. Am J Nephrol 2004;24:198–211. 14988643 10.1159/000077065

[24] Liu Y Zhao P Qu Z Frequency of CD4+CXCR5+ TFH cells in patients with hepatitis b virus-associated membranous nephropathy. Int Immunopharmacol 2014;22:98–106. 24975830 10.1016/j.intimp.2014.06.024

[25] Li Y Ma S Tang L Circulating chemokine (C-X-C Motif) receptor 5 (+) CD4 (+) T cells benefit hepatitis B e antigen seroconversion through IL-21 in patients with chronic hepatitis B virus infection. Hepatology 2013;58:1277–86. 23703545 10.1002/hep.26489

[26] Gu J Sun R Shen S The curative effect of adefovir dipivoxil treating HBeAg negative chronic hepatitis B and treating HBeAg positive chronic hepatitis B combining interferon (-2b. Pak J Pharm Sci 2015;28 (4 suppl):1493–7. 26431662

[27] Shen X Fu B Liu Y NKp30 (+) NK cells are associated with HBV control during pegylated-interferon-alpha-2b therapy of chronic hepatitis B. Sci Rep 2016;6:38778. 27941937 10.1038/srep38778 PMC5150634

